# Evaluating the Aqueous Phase From Hydrothermal Carbonization of Cow Manure Digestate as Possible Fertilizer Solution for Plant Growth

**DOI:** 10.3389/fpls.2021.687434

**Published:** 2021-06-30

**Authors:** Silvia Celletti, Maximilian Lanz, Alex Bergamo, Vittoria Benedetti, Daniele Basso, Marco Baratieri, Stefano Cesco, Tanja Mimmo

**Affiliations:** ^1^Faculty of Science and Technology, Free University of Bolzano-Bozen, Bolzano, Italy; ^2^HBI S.r.l., Bolzano, Italy; ^3^Competence Centre for Plant Health, Free University of Bolzano-Bozen, Bolzano, Italy

**Keywords:** digestate management, hydrothermal carbonization, liquid phase, maize, nutrients, sustainability

## Abstract

Improving the agronomic use of recycled nutrients derived from organic waste is one of the priorities within the measures adopted by the European community to reduce environmental issues but remains an unexplored area of research. This study focused on investigating the possibility of using innovative fertilizer solutions in hydroponic systems for the growth of agricultural plants. To this purpose, a liquid fraction [aqueous hydrothermal carbonization (HTC) liquid (AHL)] derived from HTC of cow manure digestate was chemically characterized (pH, electrical conductivity, mineral elements, and organic compounds such as phytotoxins), diluted with distilled water (1:30, 1:60, and 1:90, v/v) to reduce its potential phytotoxicity, and used to grow hydroponic maize (*Zea mays* L.) plants instead of the classical full-strength nutrient solution. The results indicated that the dilution ratio 1:30 of the AHL solution maintained a high level of toxicity for the plants (phytotoxic substances, especially Na and alkalinity), inducing the arrest of their growth. Differently, the two other dilution ratios (i.e., 1:60 and 1:90) seemed to considerably limit the levels of toxicity, since they allowed the plants to develop. However, these dilution ratios were poor in nutrient elements, inducing alteration in photosynthesis and an onset of deficiency symptoms such as pronounced leaf chlorosis. In view of an eco-friendly approach, future studies are, therefore, needed to identify the correct species-specific dilution ratio to supply both low levels of phytotoxins and adequate content of essential nutrients for appropriate plant growth and development. Furthermore, in order to lower specific Na phytotoxicity, treatments are of utmost importance before using AHL as a fertilizer solution.

## Introduction

There is a clear need for waste management actions aimed at encouraging restraint of waste volumes and efficient recovery and use of resources still present in waste (Directive 2006/12/EC, 2006). Anaerobic digestion (AD) is a biological process applied to biodegradable wastes (e.g., animal manure, sewage sludge, organic fraction of municipal solid waste, and aquaculture residues) for their conversion into biogas to be exploited for energy purposes (Weiland, 2010; Appels et al., 2011). In the context of natural resource recycling and reuse, digestate, the solid/liquid by-product of AD, should also be valorized. Although digestate is used in agriculture for its high mineral nutrient content [mainly nitrogen (N), phosphorus (P), and potassium (K)] and organic matter (Baştabak and Koçar, 2020), it can be considered as an effective organic soil amendment or fertilizer only if managed properly (Nkoa, 2014). In this respect, it is important to highlight that N input must be limited to 170 kg ha^−1^ year^−1^ on agricultural soils in vulnerable areas according to European Directives 2016/2284/EU and 91/676/EEC (Celletti et al., 2021). Consequently, in specific agricultural contexts/areas, digestate must be disposed of as waste with relevant operational transport and energy consumption for drying (mainly related to its high water content that can reach up to 90–95%, w/w) and, subsequently, additional environmental costs (Timonen et al., 2019).

An innovative technological solution, proposed to treat and valorize digestate and wet biomass in general, is represented by hydrothermal carbonization (HTC; Mumme et al., 2011; Hitzl et al., 2015; Pecchi and Baratieri, 2019). Unlike conventional dry thermochemical processes (e.g., combustion, pyrolysis, and gasification), HTC does not require an expensive or energy-intensive preliminary drying step, as it directly exploits the water retained in digestate as a solvent during the process. In particular, the HTC process takes place between 180 and 250°C and 10 and 80 bars, with residence time ranging from few minutes to several hours. The HTC process converts wet feedstock into gas (mainly CO_2_), a carbonaceous solid fraction (termed hydrochar), and a liquid phase [termed aqueous HTC liquid (AHL); Funke and Ziegler, 2010; Maniscalco et al., 2020].

In the last 20 years, hydrochar has gained attention because of its chemical and physical properties, which can be exploited for numerous purposes (Hu et al., 2008; Fang et al., 2018). For instance, hydrochar can be used as (i) solid fuel for energy production (Lucian and Fiori, 2017); (ii) adsorbent material to remediate polluted soils and water because of toxic substances (Elaigwu et al., 2014; Fornes et al., 2015; Fang et al., 2018; Parlavecchia et al., 2020); and (iii) organic soil amendment, being rich in carbon (C) and nutrients (Maniscalco et al., 2020). Recently, it has also been suggested as either a stand-alone substrate or a constituent of growing media for plants in soilless culture systems (Belda et al., 2016; Gruda, 2019). However, the evaluation of the effectiveness of hydrochar in the aforementioned applications is still ongoing, since it depends on the initial biomass type/composition and on operating parameters (i.e., temperature, residence time, and pressure).

The valorization of AHL is much more challenging than that of hydrochar, and the literature regarding the knowledge at the level of chemical characterization and subsequent possible applications of AHL is remarkably scarce (Vozhdayev et al., 2015; Mau et al., 2019; Langone and Basso, 2020). Similar to hydrochar, the differences in the chemical compositions of AHL depend on the type/composition of the feedstock and operating conditions adopted during HTC, which affects the distribution of compounds between the solid and liquid phases (Langone and Basso, 2020).

Overall, AHL is naturally composed of water and a mixture of organic and inorganic compounds (Lucian and Fiori, 2017): organic acids, carbohydrates, nutrients (especially nitrogen and phosphorus), dissolved salts, and heavy metals (Nakhshiniev et al., 2014; Huang and Yuan, 2016). In addition, most potentially phytotoxic organic compounds (mainly furan derivatives and aromatic compounds; Bargmann et al., 2013; Puccini et al., 2018; Celletti et al., 2021), which are formed during the HTC process from biomass polymers (Funke and Ziegler, 2010), are water-soluble and, therefore, concentrated in the AHL (Karagöz et al., 2005; Bargmann et al., 2013; Elaigwu and Greenway, 2016).

Some routes have been suggested for AHL exploitation as a recirculation substrate in a closed-loop system for AD (Pecchi and Baratieri, 2019) or HTC (Stemann et al., 2013; Weiner et al., 2014; Catalkopru et al., 2017) to reduce large AHL volumes. Alternatively, considering its high nutrient content, AHL can be used as a nutrient source for microalgae growth (Levine et al., 2013; Belete et al., 2019), and for irrigation in agricultural fields (Nicolae et al., 2020), fertilizer production (Yahav Spitzer et al., 2018; Wang et al., 2019), or recovery of chemicals such as N and P (Becker et al., 2019; Ovsyannikova et al., 2019).

Among the few studies that evaluated the possibility of valorizing AHL using it as a fertilizer for plant species, several other ones considered the effects of AHL on plant growth by mixing it with hydrochar or other organic substrates (Busch et al., 2013; Jandl et al., 2013; Sun et al., 2014). To the best of the knowledge of the authors, only two studies (Vozhdayev et al., 2015; Mau et al., 2019) investigated the effect of AHL alone on plant growth as a liquid fertilizer added to inorganic substrates (e.g., silica or quartz sand), showing, in some cases, inhibitory and, in others, stimulatory plant responses according to the origin and levels of AHL applied. However, none of these studies examined plant growth responses using the AHL solution alone as a substitute for the nutrient solution commonly adopted in soilless systems. This study is the first to fill this knowledge gap. Specifically, it aims to evaluate the possibility of using AHL from cow manure digestate by testing it at three different dilution ratios (1:30, 1:60, and 1:90, v/v) with distilled water as a possible nutrient solution to support the hydroponic growth of maize (*Zea mays* L.), which is selected because of its fast growth.

## Materials and Methods

### Digestate Collection, Moisture, and Microbiological Analysis

Semi-liquid cow manure anaerobic digestate was used as feedstock for the HTC process. It was provided by the company “Biogas Wipptal Srl,” located in Vipiteno, Italy.^1^

Total moisture content of the digestate was determined in accordance with ISO 18134-3:2015 (EN ISO 18134-3:2015, 2015). Briefly, the fresh digestate was weighed, placed in aluminum crucibles inside an oven, and heated up to 105°C for 24 h. Subsequently, it was cooled in a desiccator and weighed again to calculate the moisture content.

Detection and enumeration of fecal coliforms (*Escherichia coli*) and Salmonella spp. in the digestate were performed according to the procedure ISO 7251:2005 (ISO 7251:2005, 2005) and USEPA 1682:2006 [USEPA (U.S. Environmental Protection Agency), 2006].

### Chemical Characterization of Digestate

Prior to chemical analyses, the digestate was oven-dried at 105°C until a constant weight was reached, and then it was finely pulverized and homogenized with a ball mill (Mixer Mill, MM400, RETSCH, Bergamo, Italy).

The pH and electrical conductivity (EC) of the water extract were measured by immersing the electrode of a portable pH meter (pH 70 + DHS, Giorgio·Bormac Srl, Modena, Italy) and a conductivity meter (EC-meter, edge™ HI2020-02, HANNA Instruments Srl, Salerno, Italy), respectively. The water extract was obtained from 2-g dry weight (DW) of pulverized digestate diluted in 40 ml Milli-Q water (1:20, w/v), after 30 min of shaking and 5 min of centrifugation at 4,000 rpm at room temperature.

Total C and total N contents were determined by weighting approximately 2.5 mg DW of pulverized digestate into tin capsules (5 × 9 mm, Säntis Analytical AG, Teufen, Switzerland), carefully closed with tweezers and inserted into the sample tray of a Flash Elemental Analyzer (Flash EA 1112, ThermoFisher Scientific, Dreieich, Germany). The Flash EA had an oxidation furnace temperature of 1,020°C and a reduction furnace temperature of 900°C. In an integrated gas chromatograph, the gas mixture was separated and measured by means of a thermal conductivity detector. The results were expressed as a percentage of C and N.

The concentrations of the main mineral elements were determined by mineralizing approximately 0.3 g DW of pulverized digestate with 4 ml of concentrated ultrapure nitric acid (HNO_3_, 650 ml L^−1^, Carlo Erba, Milan, Italy), using a single reaction chamber microwave digestion system (UltraWAVE, Milestone, Shelton, CT, United States). After cooling, the digested sample was diluted with Milli-Q water to 20 ml. Finally, the concentrations of the elements were analyzed with an inductively coupled plasma-optical emission spectroscopy (ICP-OES, Spectro Arcos, Spectro Ametek, Kleve, Germany) instrument using spinach leaves (SRM 1570a) and tomato leaves (SRM 1573a) as external certified reference materials.

The ash content of the digestate was determined in accordance with EN ISO 18122:2015 (EN ISO 18122:2015, 2015). Briefly, the dry and pulverized digestate was weighed and then placed in ceramic crucibles inside a muffle furnace. It was heated according to the following temperature program: (a) ramp from room temperature to 250°C at 5°C min^−1^; (b) hold at 250°C for 60 min; (c) ramp to 550°C at 10°C min^−1^; (d) hold at 550°C for 120 min; (e) allow the temperature to drop to 105°C, and (f) hold at 105°C until it was removed. After the digestate was taken out of the muffle, it was cooled in a desiccator and weighed again to calculate the ash content.

### Hydrothermal Carbonization Experiment and Sampling of AHL

Hydrothermal carbonization of the digestate was conducted in a 4-L stainless steel batch reactor in order to produce the liquid fraction (AHL). The detailed description of the experimental procedure is reported elsewhere (Celletti et al., 2021). Briefly, 2 L of the digestate, previously stored at 4°C, was placed in the reactor and heated up to 180°C at a rate of 4°C min^−1^. The set process temperature was kept constant for a residence time of 3 h, and then the reactor was left to cool overnight. The latter was opened at room temperature to collect the resulting semi-solid product. Subsequently, AHL was obtained by separating it from the solid fraction (hydrochar) by centrifugation at 5,000 rpm for 5 min at 4°C and collected in dark glass bottles. The AHL was stored at 4°C before being chemically characterized and used in the plant experiment.

### Chemical Characterization of AHL

The AHL was thoroughly vortexed prior to being used for chemical characterization.

The pH and electrical conductivity (EC) of the AHL were measured directly by immersing the electrode of a portable pH-meter (pH 70 + DHS, Giorgio·Bormac Srl, Modena, Italy) and a conductivity meter (EC-meter, edge™ HI2020-02, HANNA Instruments Srl, Salerno, Italy), respectively.

The total organic carbon (TOC) and total nitrogen (TN) of the AHL were simultaneously measured with TOC-L Analyzer equipped with TNM-L TN Unit and ASI-L Autosampler (Shimadzu Corporation, Kyoto, Japan). This apparatus adopts the 680°C combustion catalytic oxidation method to also efficiently oxidize hard-to-decompose insoluble and macromolecular organic compounds. Before inserting the aqueous phase in the autosampler collector, it was filtered through 0.45-μm regenerated cellulose syringe filters (Phenex™-RC 26 mm 0.45 u, Phenomenex, Castel Maggiore, Bologna, Italy). Afterward, the filtrates were diluted 1:200 (v/v) with Milli-Q water to a final volume of 8 ml.

Two milliliter of AHL was mineralized with 2 ml of concentrated ultrapure HNO_3_ (650 ml L^−1^; Carlo Erba, Milan, Italy), using a single reaction chamber microwave digestion system (UltraWAVE, Milestone, Shelton, CT, United States). After cooling, the digested sample was diluted with Milli-Q water to 20 ml. Finally, the concentrations of the main mineral elements were determined by ICP-OES (Spectro Arcos, Spectro Ametek, Kleve, Germany) analysis, using spinach leaves (SRM 1570a) and tomato leaves (SRM 1573a) as external certified reference materials.

Sugars (glucose), organic acids (lactic acid, formic acid, acetic acid, and fumaric acid), and furan compounds [5-hydroxymethylfurfural (HMF) and furfural] in the AHL were separated and quantified simultaneously by high performance liquid chromatography (HPLC) using a cation exchange column Aminex 87-H column (300 × 7.8 mm, 9 μm, Bio-Rad Laboratories, Segrate, Milano) and an isocratic elution with 10 mM H_2_SO_4_ as a carrier solution at a flow rate of 0.6 ml min^−1^. Sugars were detected with a refractive index detector (2414 RI, Waters Spa, Milan, Italy), while organic acids and furan compounds were detected at 210 and 280 nm, respectively, using a photodiode array detector (2998 PDA, Waters Spa, Milan, Italy). Standards for each analyte were prepared as individual stock solutions, using reagent-grade compounds (Merck, Darmstadt, Germany) and then combined to give diluted reference standards. All compounds were identified by comparing retention times of unknowns to pure compounds.

Each analysis was repeated three times (technical replicates, *n* = 3).

### Plant Growth

Maize (*Z. mays* L) seeds were soaked in distilled water for 24 h. Subsequently, they were transferred on a narrow mesh net placed in a container with an aerated solution of 0.5 mmol L^−1^ CaSO_4_ and left to germinate for 6 days in the dark at room temperature. Six-day-old seedlings were selected on the basis of their size uniformity to be transferred into 1.5-L plastic pots (10 seedlings/pot) filled with a continuously aerated nutrient solution (NS) with the following composition (mM): Ca(NO_3_)_2_x 4H_2_O 2, KCl 0.1, KH_2_PO_4_ 0.1, K_2_SO_4_ 0.7, MgSO_4_x 7H_2_O 0.5, CuSO_4_x 5H_2_O 0.2 × 10^−3^, Fe(III) – EDTA 0.1, H_3_BO_3_ 1 × 10^−3^, MnSO_4_x H_2_O 0.5 × 10^−3^ (NH_4_)_6_Mo_7_O_24_x 4H_2_O 0.01 × 10^−3^, and ZnSO_4_x 7H_2_O 0.5 × 10^−3^ (slightly modified by Zhang et al., 1991). The pH of the NS was titrated at 6 with 0.1 mM MES-KOH. After 3 days of growing in the NS, the maize plants were transferred into aerated solutions of the AHL diluted with distilled water at a ratio of 1:30, 1:60, and 1:90 (v/v) and grown for additional 12 days. The control plants were grown simultaneously using the NS instead of the AHL. The plants were cultivated for a total of 15 days in a growth chamber under controlled climatic conditions with a day/night cycle of 14/10 h, temperature regime of 24/19°C, light intensity of 250 μmol m^−2^ s^−1^, and relative humidity of 70%. Growing solutions were changed twice a week, and the pots were rotated randomly to a different position within the block each day for the duration of the experiment.

### Measurement of Plant Growth and Root Morphological Parameters

At the end of the experimental growing period (15 days after sowing), the leaf chlorophyll content of the maize plants was measured using a portable non-destructive tool, the Soil Plant Analysis Development (SPAD – 502 Plus, Konica Minolta, Osaka, Japan). Specifically, three SPAD values were taken from the base to the apex (along the proximal, central, and distal portions) of the youngest fully expanded leaf of each plant, resulting in a total of 36 measurements (12 plants × 3 repeats) per treatment, and were averaged and expressed as SPAD index. Subsequently, the maize plants were harvested by separating shoots from roots. The roots were gently rinsed with distilled water. Shoot and root fresh weights (FWs) were recorded, and root-to-shoot ratios were assessed. Digital scans of the root morphological and architectural features (i.e., total length and number of tips) were analyzed with a WinRHIZO™ system (EPSON1680, WinRHIZO Pro2003b Software; Regent Instruments Inc., Quebec, Canada). The roots and shoots were consequently dried at 65°C until a constant weight was reached.

### Analysis of Main Essential Nutrients and Sodium in Plant Tissues

Dried plant tissues were finely ground and homogenized with a ball mill (Mixer Mill, MM400, RETSCH, Bologna, Italy). Approximately 0.3 g DW of each ground sample was mineralized in 4 ml concentrated ultrapure HNO_3_ (650 ml L^−1^; Carlo Erba, Milan, Italy) using a single reaction chamber microwave digestion system (UltraWAVE, Milestone, Shelton, CT, United States). After cooling, the digested samples were diluted with Milli-Q water to 20 ml and analyzed by ICP-OES (Spectro Arcos, Spectro Ametek, Kleve, Germany) as previously described.

### Statistical Analysis

The data are expressed as means ± SE of technical replicates (*n* = 3) for both the digestate and the AHL analysis and of biological replicates (*n* = 12) for plant analysis. The significance of differences among the means was calculated by one-way ANOVA with LSD test at *p* < 0.05 using the R software (version 3.6.3). R packages, ggplot2 (version 3.3.2), and agricolae (version 1.3-3) were used for data visualization and statistical analysis.

## Results

### Digestate Properties

Table 1 includes the physical (i.e., moisture content) and microbiological (i.e., *E. coli* and Salmonella spp.) parameters of the digestate, which was used as feedstock for the HTC process. The digestate had a relatively high moisture content (approximately 90% on FW basis) and had, therefore, a semi-liquid consistency, and it did not contain fecal bacteria potentially harmful to human health and the environment. Indeed, the number of *E. coli* (on DW basis) resulted in less than 3, i.e., below the limit of quantification of the method, and Salmonella species (on FW basis) were absent (Table 1).

**Table 1:** Physical and microbiological properties of the digestate.

Parameter	
Moisture (%, w/w)	90.34 ± 0.04
*Escherichia coli* (MPN/g_DW_)	<3
Salmonella spp. (MPN/25g_as-is_)	Absent

MPN, most probable number; DW, dry weight.

**Figure 1:**
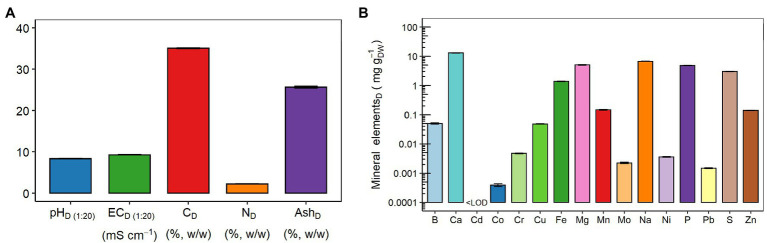
Chemical properties of the digestate (D): **(A)** pH, electrical conductivity (EC), carbon (C), nitrogen (N), and ash contents; and **(B)** mineral element concentrations: boron (B), calcium (Ca), cadmium (Cd), cobalt (Co), chromium (Cr), copper (Cu), iron (Fe), magnesium (Mg), manganese (Mn), molybdenum (Mo), sodium (Na), nickel (Ni), phosphorus (P), lead (Pb), sulfur (S), and zinc (Zn). LOD, limit of detection. The LOD of Cd was 0.177 μg L^−1^. Data are presented as means ± SE (*n* = 3). Data of mineral element concentrations are presented on a logarithmic scale (Log_10_) for better graphical display, and the values are detailed in Supplementary Table 1.

Figure 1A shows that the digestate was characterized by alkaline pH_(1:20)_ (8.37 ± 0.03), EC_(1:20)_ value of 9.27 ± 0.06 mS cm^−1^, and contents of total C, total N, and ash (in percentage on DW basis) were 35.07 ± 0.10, 2.27 ± 0.04, and 25.64 ± 0.19, respectively. Figure 1B shows the composition and total concentration of each of the 16 mineral elements detected in the dried digestate. Specifically, the concentration values of these elements are detailed in Supplementary Table 1. The concentration of Ca (13 ± 0.3 mg g_DW_^−1^) prevailed over the remaining macronutrients, such as Mg > P > and S, whose levels ranged from 3 to 6 mg g_DW_^−1^ (Figure 1B; Supplementary Table 1). Among the micronutrients (i.e., B, Cu, Fe, Mn, Mo, and Zn), the highest concentration was observed for Fe (1.4 ± 0.1 mg g_DW_^−1^), followed by Mn > Zn > B ≈ Cu >> Mo (Figure 1B; Supplementary Table 1). Regarding the heavy metals (i.e., Cd, Co, Cr, Ni, and Pb), Cr was the most abundant (4.7 ± 0.2 μg g_DW_^−1^), whereas the Cd concentration was below the limit of detection (LOD = 0.177 μg L^−1^; Figure 1B; Supplementary Table 1). Finally, Na content was equal to 6.7 ± 0.1 mg g_DW_^−1^ (Figure 1B; Supplementary Table 1).

### AHL Properties

**Figure 2:**
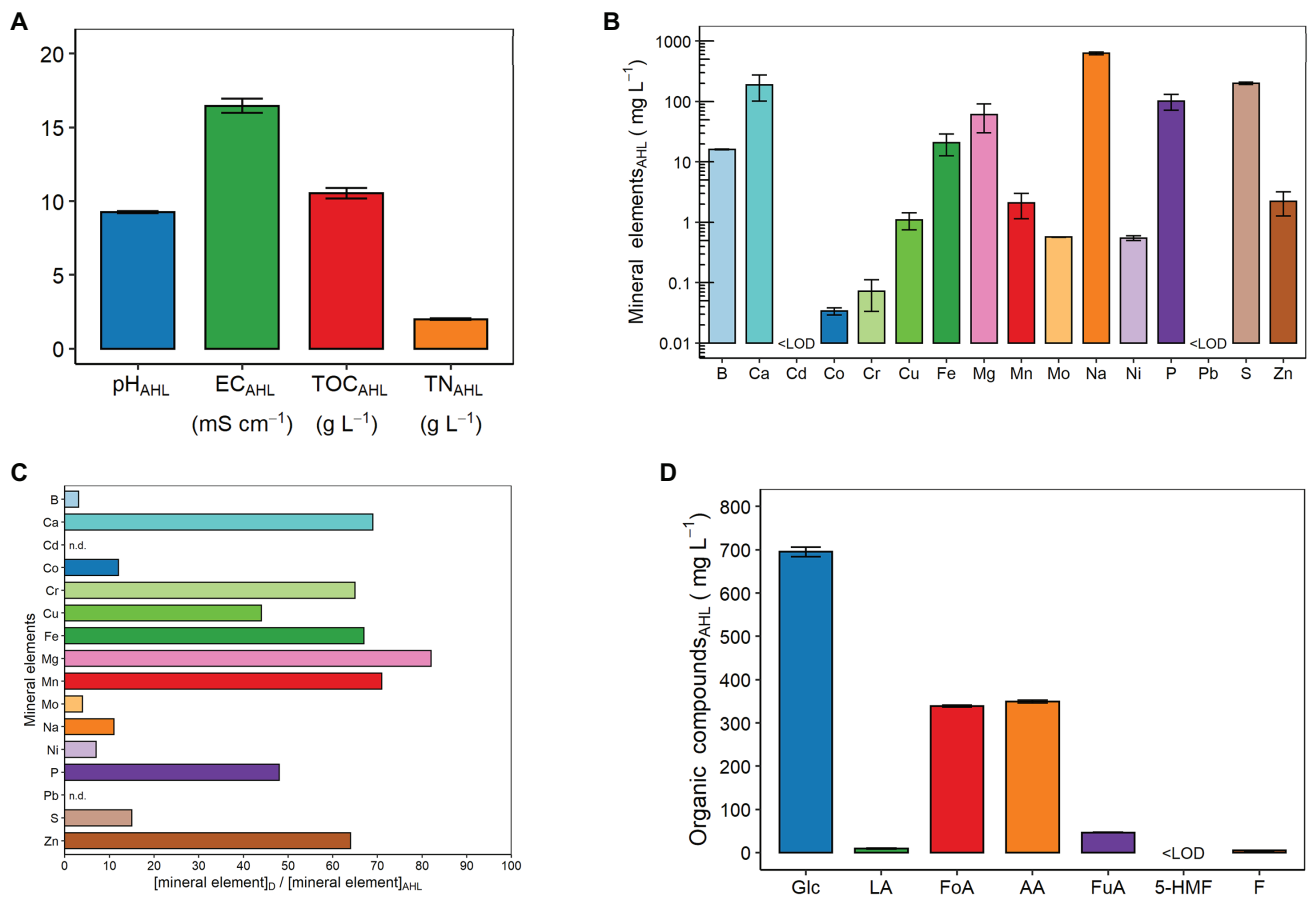
Chemical properties of the aqueous liquid fraction of hydrothermal carbonization [aqueous HTC liquid (AHL)]: **(A)** pH, electrical conductivity (EC), total organic carbon (TOC), and total nitrogen (TN) content; **(B)** mineral element concentrations: boron (B), calcium (Ca), cadmium (Cd), cobalt (Co), chromium (Cr), copper (Cu), iron (Fe), magnesium (Mg), manganese (Mn), molybdenum (Mo), sodium (Na), nickel (Ni), phosphorus (P), lead (Pb), sulfur (S), and zinc (Zn); **(C)** ratio between the concentration in the digestate (D) and the concentration in the AHL of each of the following mineral elements: boron (B), calcium (Ca), cadmium (Cd), cobalt (Co), chromium (Cr), copper (Cu), iron (Fe), magnesium (Mg), manganese (Mn), molybdenum (Mo), sodium (Na), nickel (Ni), phosphorus (P), lead (Pb), sulfur (S), and zinc (Zn). For Cd and Pb, the ratio was not determinable (n.d.), as the mineral element concentration was below the LOD (i.e., 0.177 and 6.730 μg L^−1^, respectively) in D and/or AHL; and **(D)** organic compound concentrations: glucose (Glc), lactic acid (LA), formic acid (FoA), acetic acid (AA), fumaric acid (FuA), 5-hydroxymethylfurfural (5-HMF), and furfural (F). LOD, limit of detection. The LODs of Cd and Pb by inductively coupled plasma-optical emission spectroscopy (ICP-OES) were 0.177 and 6.730 μg L^−1^, respectively. The LOD of 5-HMF was 0.706 mg L^−1^ and was determined using the method described by Miller and Miller (2010). Data are presented as means ± SE (*n* = 3). Data of mineral element concentrations are presented on a logarithmic scale (Log_10_) for better graphical display, and the values are detailed in Supplementary Table 2.

Figures 2A,B show the same chemical parameters examined for the digestate. As observed earlier for the digestate, the pH of the AHL was alkaline (9.24 ± 0.07; Figure 2A). In particular, the HTC process increased the pH by one unit compared with the digestate (Figure 1A). In addition, the process also increased the EC value (i.e., the concentration of dissolved ions and salts), which was approximately twice that of the digestate and equal to 16.46 ± 0.47 mS cm^−1^ (Figure 2A). Moreover, the AHL had TOC and TN contents of 10.53 ± 0.35 and 1.99 ± 0.05 g L^−1^, respectively (Figure 2A). Thus, with the HTC process, the C content was reduced by about slightly more than three times, while the N content remained almost constant (Figure 2A). Overall, as for the digestate, the C content in the AHL was always higher than the N content (Figures 1A, 2A). Specifically, the TOC was five and 15 times higher than the TN content in the AHL (Figure 2A) and the digestate (Figure 1A), respectively. Figure 2B displays the composition and total concentration of the 16 mineral elements also analyzed in the digestate. Specifically, the concentration values of these elements are detailed in Supplementary Table 2. Among the macronutrients (i.e., Ca, Mg, P, and S), the S concentration in the AHL was the highest (200.3 ± 10.1 mg L^−1^), followed closely by Ca and then in the following order by P > Mg (Figure 2B; Supplementary Table 2). With regard to the micronutrient (B, Cu, Fe, Mn, Mo, and Zn) contents, Fe was the most abundant (20.8 ± 8.3 mg L^−1^) and Mo (570 ± 5.0 μg L^−1^) was the least abundant as observed in the digestate. In particular, the B content was close to that of Fe (16.2 ± 0.2 mg L^−1^), while Zn, Mn, and Cu contents varied between approximately 2 and 1 mg L^−1^ (Figure 2B; Supplementary Table 2). Among the heavy metals (i.e., Cd, Co, Cr, Ni, and Pb), unlike in the digestate, the most abundant was Ni (546 ± 49.1 μg L^−1^) and then in the following order Cr > Co, with the Cd and Pb contents below LOD (0.177 and 6.730 μg L^−1^, respectively). Specifically, the Cd content was lower than LOD not only in the AHL (Figure 2B; Supplementary Table 2) but also in the digestate (Figure 1B). Finally, the Na content was equal to 628.3 ± 34.2 mg L^−1^, and this value was even greatly higher than that of the macronutrients measured in the AHL (Figure 2B; Supplementary Table 2).

In order to evaluate the effect of the HTC process on the distribution of nutrients between the liquid and solid phases, the ratio between the concentration in the digestate and the concentration in the AHL of the 16 mineral elements has been measured (Figure 2C). Apart from Cd, which was not detected by the ICP-OES analysis of the digestate and, therefore, obviously not in the AHL, in general, all the element concentrations decreased from the digestate to the AHL, although with very different variations (Figure 2C). Specifically, two groups, in terms of the extent of reduction, can be clearly distinguished: the first group, with Mg > Mn > Ca > Fe > Cr > Zn > P > Cu, showed reductions varying from 82 to 44 times, and the second group, with S > Co > Na > Ni > Mo > B, showed reductions varying from 15 to three times (Figure 2C). On the other hand, Pb was present in the digestate (1.5 ± 0.1 μg g_DW_^−1^), but in the AHL, its concentration was it lower than that of instrumental LOD (Figure 2C).

Figure 2D shows the seven organic compounds identified within the AHL, namely, sugars [i.e., glucose (Glc)], acids [i.e., lactic (LA), formic (FoA), acetic (AA), and fumaric (FuA)], and furans [i.e., 5-hydroxymethylfurfural (5-HMF) and furfural (F)]. The most abundant was Glc (695.07 ± 10.64 mg L^−1^; Figure 2D). Among the four organic acids identified in the AHL, the concentration of AA prevailed, being 349.14 ± 3.33 mg L^−1^. However, this concentration was not much higher than that of FoA, which reached a concentration of 338.86 ± 2.60 mg L^−1^. In contrast, these concentrations were about seven times higher than that of FuA and about 38 times higher than that of LA. In particular, the concentrations of FuA and LA were 46.31 ± 0.64 and 9.19 ± 0.87 mg L^−1^, respectively (Figure 2D). In the case of the two furan compounds, a higher concentration was observed for F (4.87 ± 0.22 mg L^−1^), since the 5-HMF concentration was below LOD (0.706 mg L^−1^) in the AHL (Figure 2D).

### Effect of Different AHL Dilution Ratios on Maize Plant Growth and Related Traits

**Figure 3:**
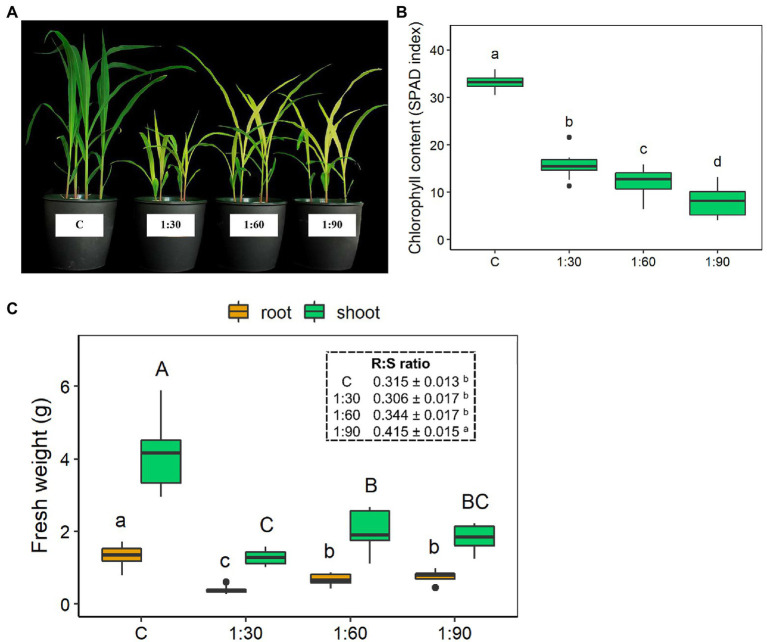
Growth parameters of 15-day-old maize seedlings grown in the full-strength nutrient solution (C, control) and in the AHL diluted with distilled water in a ratio of 1:30, 1:60, and 1:90 (v/v): **(A)** representative shoots, **(B)** leaf chlorophyll content, and **(C)** shoot and root fresh weight (FW). Insert: root (R) to shoot (S) ratio. Data are presented as means ± SE (*n* = 12). Statistical significance was tested by one-way ANOVA analysis with LSD post-test (*p* < 0.05). Statistically significant differences among the four different growth conditions are indicated by different letters: different lower case letters indicate significant differences among the growth conditions in roots; different upper case letters indicate significant differences among the growth conditions in shoots.

Significant differences in leaf color and growth were visually evident in the 15-day-old maize plants grown either with the full-strength NS (control condition – C) or with the three different diluted AHL solutions (1:30, 1:60, and 1:90; Figure 3A). These visual differences were confirmed by the leaf chlorophyll contents (Figure 3B) and biomass accumulation (i.e., fresh weight) of both the shoots and the roots (Figure 3C). None of the three AHL dilution ratios allowed the plants to achieve chlorophyll contents similar or equivalent to that of the control plants (33.2 ± 0.5 SPAD index; Figure 3B). Moreover, this reductive effect on chlorophyll content was significantly more pronounced as the dilution ratio decreased (1:30 > 1:60 > 1:90). Indeed, the chlorophyll content for 1:30 plants was reduced by about half, for 1:60 plants by almost 3-fold, and for 1:90 plants by slightly more than 4-fold when compared with the control (Figure 3B). The control plants also exhibited the highest fresh weight at both shoot and root levels compared with the plants grown with the diluted AHL solutions (Figure 3C). In this case, the 1:30 treatment had the most dramatic reducing effect on the shoot and root fresh biomass accumulation (−70% vs. C in both shoots and roots) than the 1:60 and 1:90 treatments (Figure 3C). However, the root:shoot ratio increased by 32% only with the 1:90 dilution level, as no statistically significant changes were observed for the two other treatments compared with the control (insert of Figure 3C). Among the three different AHL dilution ratios, there was a 35% decrease in shoot fresh weight only for 1:30-treated compared with the 1:60-treated plants, whereas the shoot fresh weight of the 1:90-treated plants did not show significant differences from those of the 1:30- and 1:60-treated plants. On the other hand, the root fresh weights of the 1:60- and 1:90-treated plants did not differ statistically from each other, but both were significantly higher (+88%) than that of the 1:30-treated plants (Figure 3C).

**Figure 4:**
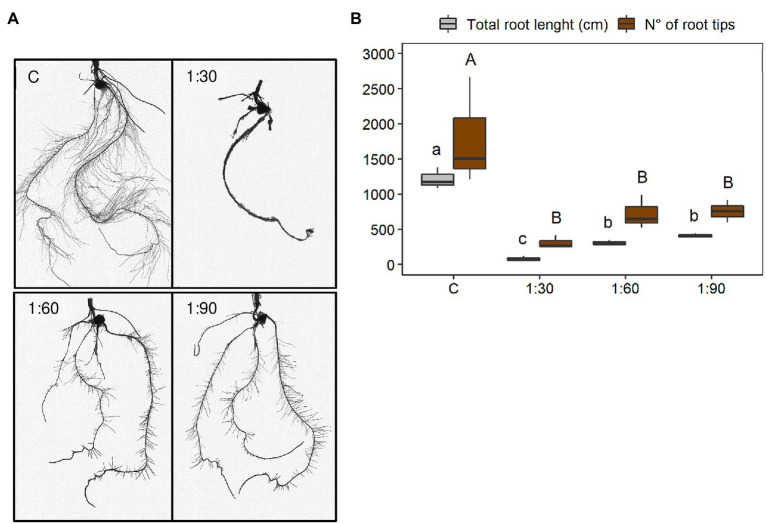
Morphological parameters of root systems of 15-day-old maize seedlings grown in the full-strength nutrient solution (C, control) and in the AHL diluted with distilled water in a ratio of 1:30, 1:60, and 1:90 (v/v): **(A)** WinRHIZO images and **(B)** total root length and number of root tips. Data are presented as means ± SE (*n* = 12). Statistical significance was tested by one-way ANOVA analysis with LSD post-test (p < 0.05). Statistically significant differences among the four different growth conditions are indicated by different letters: different lower case letters indicate significant differences among the growth conditions in total root length; different upper case letters indicate significant differences among the growth conditions in a number of root tips.

At the root morphological level, significant visual differences were also clearly distinguished among the four different growth conditions of the maize plants (Figure 4A). Visually, the root systems of the treated plants increased with increasing dilution ratio and resulted more similar to the control. However, the treated plants had a less developed root system, especially in terms of lateral root length (Figure 4A). These differences were confirmed by two morphological parameters such as total root length and number of root tips (Figure 4B). Overall, both parameters were greatly reduced in plants grown with the AHL compared with the control plants (C). In particular, there were no significant differences in total root length between the 1:60 and 1:90 treatments, whereas the effect of the 1:30 treatment was remarkably evident on the reduction of this parameter (−74% vs. 1:60 and −80% vs. 1:90; Figure 4B). For the number of root tips, no statistically significant change was observed between the 1:30, 1:60, and 1:90 treatments, although a reduction to a greater extent was observed for the 1:30 treatment (−57% vs. 1:60 and −59% vs. 1:90; Figure 4B). Accordingly, the reductions in both morphological parameters were clearly sharpened especially when comparing plants supplemented with the most concentrated AHL (i.e., 1:30-treated plants) with the control (−93% for total root length and −83% for number of root tips; Figure 4B).

**Figure 5:**
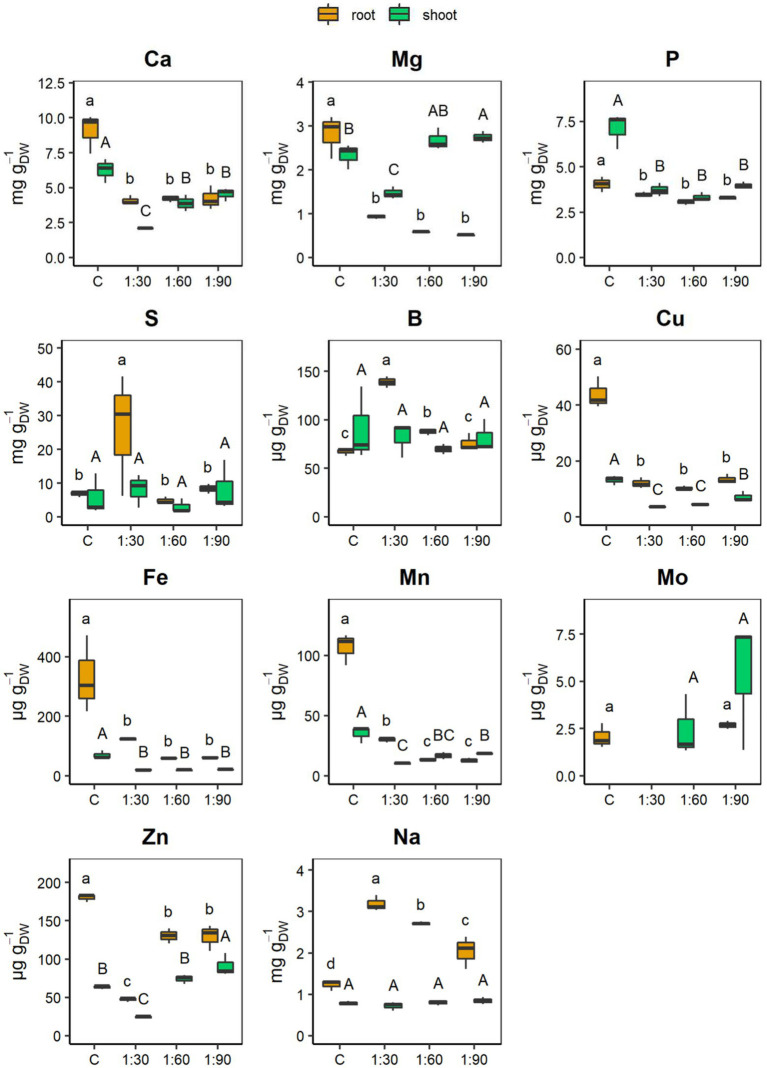
Concentrations of main macro- [calcium (Ca), magnesium (Mg), phosphorus (P), and sulfur (S)] and micronutrients [boron (B), copper (Cu), iron (Fe), manganese (Mn), molybdenum (Mo), and zinc (Zn)], and sodium (Na) in roots and shoots of 15-day-old maize seedlings grown with the full-strength nutrient solution (C, control) and with the AHL diluted with distilled water in a ratio of 1:30, 1:60, and 1:90 (v/v). Boxplots were omitted when the concentration values are below the limit of detection (LOD). The LOD of Mo was 10.107 μg L^−1^. Data are presented as means ± SE (*n* = 12). Statistical significance was tested by one-way ANOVA analysis with LSD post-test (*p* < 0.05). Statistically significant differences among the four different growth conditions are indicated by different letters: different lower case letters indicate significant differences among the growth conditions in roots; different upper case letters indicate significant differences among the growth conditions in shoots.

Figure 5 shows maize root and shoot concentrations of the main essential nutrients (macronutrients: Ca, Mg, P, and S; and micronutrients: B, Cu, Fe, Mn, Mo, and Zn) and of one of the beneficial non-essential elements (Na) for plant nutrition. The different dilution ratios of the AHL did not result in any significant change in the root contents of Ca, Mg, P, Cu, and Fe. However, the contents of Ca, Mg, P, Cu, and Fe were lower in the roots grown with the different AHLs than in control roots (Figure 5). In contrast, the root contents of Mn and Zn, although presenting values lower than those of the control, varied depending on the AHL dilution ratio. Specifically, Mn, which was reduced by 72% in the 1:30 condition compared with the control, was reduced even more (−88%) in the 1:60 and 1:90 treatments, both showing values equal to approximately half the value of the 1:30 treatment. Instead, the Zn content decreased by 28% in both the 1:60 and 1:90 conditions compared with the control, but the greatest reduction (−74%) occurred in the roots of plants grown with the 1:30 diluted AHL (thus the more concentrated one) when compared with the control (Figure 5). On the contrary, the roots of plants treated with 1:30 differed from all the other conditions for the highest content of S (26.1 ± 10.4 mg g_DW_^−1^), B (138.7 ± 3.4 μg g_DW_^−1^), and Na (3.2 ± 0.1 mg g_DW_^−1^) contents. In particular, the 1:60, 1:90, and control conditions did not show significant differences for the S content and the decrease was, on average, −75% compared with the 1:30 treatment. B and Na gradually decreased as the dilution ratio increased, but in the case of B, its content with the 1:90 treatment did not differ statistically from that with the control, whereas for Na, its content further decreased with the control (Figure 5). For the Mo content, the 1:30 and 1:60 conditions presented values below the detection limit (<10.107 μg L^−1^), whereas the 1:90 condition was not statistically different from that of the control.

On the other hand, in the shoots, the different dilution ratios of AHL did not affect significantly only P and Fe contents and both were lower than those measured in shoots of control plants (Figure 5). In contrast, the Ca, Cu, and Mn contents of the shoots, although presenting values lower than those of the C condition, varied according to the type of dilution ratio. Specifically, the Ca content was similar to that in the 1:60 and 1:90 conditions and decreased by 38 and 29%, respectively, compared with the control. However, the most severe reduction (−67%) was observed in the shoots of the plants grown with the 1:30 diluted AHL compared with the control shoots. Cu, which was reduced by about half in the 1:90 condition compared with the control, was reduced even more (by approximately slightly more than half) by the 1:30 and 1:60 treatments compared with the 1:90 treatment. Finally, for the Mn content, there were significant differences only between the shoots of the 1:30- and 1:90-treated plants (with 1:30 < 1:90), since the Mn content in the 1:60 treatment did not differ statistically from either the 1:30 or 1:90 treatment (Figure 5). The shoots from the 1:90-treated plants differed from those of the control in higher Mg (2.7 ± 0.1 mg g_DW_^−1^), Mo (5.4 ± 2 μg g_DW_^−1^), and Zn (90.8 ± 8.5 μg g_DW_^−1^) contents. Exactly, there was no statistically significant variation between the Mg content in the 1:60 and 1:90 shoots. In turn, however, the Mg present in the 1:60 shoots did not differ significantly from that of the control shoots. Instead, the Mg content was greatly reduced (−44%) in the 1:30 shoots compared with the 1:90 shoots. The Mo content in 1:60 statistically equaled the high levels measured in the 1:90 shoots, while it was below the detection limit (10.107 μg L^−1^) in both 1:30 and C conditions. Finally, Zn gradually decreased as the AHL concentration increased (1:90 > 1:60 > 1:30), with the 1:60 treatment exhibiting values similar to those of the control. Thus, the shoots of the 1:30-treated plants differed from all the other conditions by lowest contents not only of Ca (2.1 ± 0 mg g_DW_^−1^) but also of Mg (1.5 ± 0.1 mg g_DW_^−1^) and Zn (24.4 ± 1.0 μg g_DW_^−1^; Figure 5). Lastly, for the shoot contents of elements, such as S, B and Na, there was no statistically significant variation between the maize plants grown with the dilute AHL solutions and the control (Figure 5).

## Discussion

Considering increasing water scarcity, urbanization, and decline of arable lands due to climate change (FAO, 2017), the development and use of value-added products from wastes, such as AHLs, is a relevant aspect not only for the scientific community but also for agricultural producers (Gruda, 2019). Studies aimed at investigating the effects of AHLs by the HTC process used as agricultural liquid fertilizers have only recently begun to be conducted (Vozhdayev et al., 2015; Mau et al., 2019). Specifically, the possibility of using AHL as a fertilizer solution in soilless horticultural systems is an entirely new field of study that, therefore, needs to be explored in the upcoming future. However, it is well-known that HTC products could also have a certain level of phytotoxicity. Toxicity might be derived from the type of feedstock as well as from the formation of a variety of harmful substances because of thermochemical conditions and reactions of biomass conversion, which affect their further utilization in the real world (Fang et al., 2018; Usman et al., 2019; Celletti et al., 2021). In this study, an eco-friendly approach was followed based on the evaluation of feedstock properties (i.e., cow manure digestate) and the chemical composition of the AHL and then its impact on maize growth.

The microbiological data presented for the digestate (Table 1) clearly demonstrated that the feedstock was free of bacteria (namely, *E. coli* and Salmonella spp.) pathogenic to human beings and the environment. Therefore, if the AD process has a potential for sanitation (Seruga et al., 2020), it can be assumed that the AHL of the corresponding digestate, obtained at a HTC temperature (180°C) and a time (3 h) higher than those commonly used for heat sterilization (Block, 2000), is consequently free of hazardous microbial load. In the digestate, the values of the microbiological parameters as well as the concentrations of the heavy metals (i.e., Cd, Cr, Cu, Ni, Pb, and Zn; Table 1; Figure 2B, respectively) respected the limits set by the current European legislation for sewage sludge use in agriculture (Council Directive 86/278/EEC, 1986) and the Italian regulation for fertilizers [Mipaaf (Ministero delle politiche agricole alimentari e forestali), 2010]. As a result, the concentrations of these heavy metals together with the remaining mineral elements were further reduced with the AHL compared with the solid phase (hydrochar; Figure 2B).

In view of using AHL as a fertilizer solution, the ratio between the concentration in the digestate and the AHL of each inorganic element has provided useful indications to understand their behavior in the distribution between the AHL and hydrochar (Figure 2C). In agreement with recent observations (Sun et al., 2013; Ekpo et al., 2016), this study showed that the HTC process tended to concentrate all the elements (Figures 1B, 2B,C), such as C (Figures 1A, 2A), in the hydrochar with respect to the AHL. For this reason, hydrochar, being rich in C and nutrients, has been reported to have the potential to be used as a soil amendment (Bento et al., 2019; Kalderis et al., 2019) or as growing substrate for plants, after some operationally feasible adjustments (Roehrdanz et al., 2019; Celletti et al., 2021). The P binding to Ca, Fe, Mg, and Mn, forming insoluble phosphate salts that precipitate and accumulate in hydrochar has been extensively studied and described (Heilmann et al., 2014; Shi et al., 2014; Huang and Tang, 2015; Wang et al., 2017). This could explain why in our conditions, the AHL became mainly depleted of these essential nutrients for plants compared with the digestate (Figure 2C). Different behaviors were observed only for Cd (keeping constantly <LOD = 0.177 μg L^−1^) and N, the concentrations of which did not vary after the HTC process. Unlike some studies reporting that Pb is almost entirely released from the solid phase (Sun et al., 2013; Ekpo et al., 2016), in our case, it was the only mineral element to exhibit concentrations below LOD (i.e., <6.73 μg L^−1^) in the AHL, suggesting that it precipitates totally in the hydrochar.

Since the conditions of the HTC process further decreased the contents of heavy metals in the AHL, already below legal limits, as discussed above, this phenomenon can be considered a positive aspect. Nevertheless, all the other mineral elements were also reduced from the digestate to the AHL. The contents of the main essential plant nutrients (in decreasing order: Mg > Mn > Ca > Fe > Zn > P > Cu) were greatly reduced (in a range of 82 to 44 times; Figure 2B). On the other hand, the Na and B contents were decreased only slightly (by 11 and three times, respectively). In particular, Na was the most abundant mineral element in the AHL (Figure 2B; Supplementary Table 2).

However, comparing AHL with solutions commonly used to grow plants hydroponically, such as the Hoagland solution (Hoagland and Arnon, 1950), AHL was still more concentrated in all the essential plant nutrients, apart from Ca and Mg, which were about the same. Indeed, the AHL contained about three times more P and S, four times more Mn, seven times more Fe, 32 times more B, 44 times more Zn, 55 times more Cu, 57 times more Mo, and even about 524 times more Na than the Hoagland solution. From this, it can be clearly deduced that the main problem was related to the extremely high concentrations of Na in the AHL, not adequate for plant growth in hydroponics. The calculation of the sodium adsorption ratio (SAR), i.e., a common indicator of the suitability of water of irrigation, showed that the AHL had a value of 14.3 meq L^−1^, almost double the value considered safe (i.e., 8 meq L^−1^), in order not to cause sodicity problems (Zaman et al., 2018). In addition, the results that emerged from the preliminary experiments with the maize plants grown with AHL have suggested the need to dilute the AHL at different ratios (1:30, 1:60, and 1:90, v/v) to prevent plant death phenomena (data not shown). Therefore, these previous detrimental effects on maize plants could be most likely attributable to high EC (16.46 ± 0.47 mS cm^−1^; Figure 2A) and high Na content (628.3 ± 34.2 mg L^−1^; Figure 2B; Supplementary Table 2), rather than the presence of potentially phytotoxic furan compounds (i.e., 5-HMF and furfural) in the AHL (Figure 2D). In fact, the 5-HMF concentration was found to be below the instrumental limit of detection (LOD < 0.706 mg L^−1^), and the furfural concentration was equal to 4.87 ± 0.22 mg L^−1^, a concentration that has been shown not to inhibit the germination of seeds of cress, which is a plant species highly sensitive to the presence of phytotoxins (Celletti et al., 2021). Furthermore, the potential phytotoxicity originated by the presence of organic acids (i.e., lactic, formic, acetic, and fumaric acid) or sugars (i.e., glucose) detected in the AHL can be excluded since they are part of the exudates synthesized by plants and released from their roots into the external medium (Warren, 2016). Overall, organic acid release into the rhizosphere is a mechanism adopted by plants to cope with situations of low inorganic nutrient availability (such as Fe and P), in order to solubilize nutrients (either by acidification or chelation) and consequently to enhance their availability for root uptake (Adeleke et al., 2017).

It is well-described in the literature that Na is considered a beneficial element but not essential for plants and mainly responsible for salt stress (Gómez-Merino and Trejo-Téllez, 2018). When the salt concentration exceeds certain concentrations (e.g., 14.61 g L^−1^ NaCl for maize plants; Farooq et al., 2015), it leads to deleterious effects on plants, such as impaired growth, reduced chlorophyll content, impeded ability to acquire water supply, induced nutritional imbalances, and toxicity phenomena (Ashraf and Harris, 2013; Machado and Serralheiro, 2017). Furthermore, the salinity threshold (EC_t_) of the majority of crops is low (ranging from 1 to 2.5 mS cm^−1^; Machado and Serralheiro, 2017) and dependent on the growth stage and plant species (Kaddah and Ghowail, 1964; Celletti et al., 2021). Although maize is classified as a plant that is moderately tolerant to salinity (EC_t_ = 1.7 mS cm^−1^; Bernstein and Ayers, 1949; Kaddah and Ghowail, 1964), the EC values of the three diluted solutions of the AHL were presumably too high for its equilibrate growth and especially for its early phase of development. On one hand, as the concentration of the AHL in the fertilizer solution increased, the biomass growth and accumulation, chlorophyll content, and the number of root tips were reduced (Figures 3A–C, 4B); on the other, visual root morphological deformations were increased in the 15-day-old maize seedlings compared with those grown with the full-strength nutrient solution (control condition; Figure 4A). It is interesting to note that within the plant organs, Na was accumulated to a greater extent in the roots than in the shoots of the maize plants and increased linearly with the amount of the AHL present in the growth solutions (1:30 > 1:60 > 1:90; Figure 5). An accumulation trend similar to that of Na was also observed for B, since high concentrations of B are often associated with salinity (Del Carmen Martínez-Ballesta et al., 2008). These greater Na and B accumulations in the roots represent a survival and resistance strategy to minimize or avoid the transport of these two elements toward photosynthetic organs (Farooq et al., 2015). Moreover, this finding suggests that Na may be the primary cause of toxicity by causing morphological deformations, reducing the growth of the root systems, and consequently leading to limited development of the whole plant, interfering with the uptake and assimilation of other nutrients. Therefore, treatments regarding, for instance, the use of ion-exchange resins (Lukey et al., 1999; Subban and Gadgil, 2019) or hydro-extraction or re-crystallization methods (Sumada et al., 2018), would be required to remove undesirable dissolved salts (mainly Na) and thus to lower the phytotoxicity and to improve the fertilizing power of the AHL-containing solutions.

Besides the presence of phytotoxic compounds, pH is one of the main factors affecting the mobility of nutrients, and consequently their acquisition by plants (Fageria and Baligar, 2004). Most nutrients are optimally available to plants in the pH range of 6.5–7.5. The pH of the AHL was very strongly alkaline (9.24 ± 0.07; Ramírez-Rodríguez et al., 2007), and it can be considered as a side effect of the high Na concentration (Machado and Serralheiro, 2017). Alkaline pH limits especially the availability of micronutrients (i.e., Cu, Fe, Mn, and Zn; Rengel, 2015) to the plants but also of some macronutrients (i.e., P; Ramírez-Rodríguez et al., 2007; Gentili et al., 2018). Among the micronutrients, the only exception is Mo, which appears to be less available at acidic pH and more available under alkaline pH (Rutkowska et al., 2017).

In this study, the alkalinity of AHLs compared with the control solution (which was kept at pH 6) may have reduced the bioavailability of micronutrients, such as Cu, Fe, and Mn, and macronutrients, such as Ca and P. In fact, the maize plants grown with the solutions with AHL accumulated less of these nutrients in both shoots and roots than plants grown with the control solution (Figure 5). In turn, this phenomenon probably explains why the leaves of the plants grown with the three different diluted AHLs appeared clearly chlorotic (Figure 3A) and why their chlorophyll content (Figure 3B) was significantly reduced compared with the control plants. Indeed, leaf yellowing is a characteristic symptom of nutrient deficiencies, in this case mainly due to the simultaneous deficiencies of Fe, Cu, and Mn, which play a crucial role in the photosynthetic process (Marschner, 2012). However, it is interesting to observe that the shoots of the 1:60 and 1:90 plants were significantly richer in some essential nutrients, such as Ca, Mg, Mn, and Mo, than the shoots of the 1:30 plants; despite having grown with the two more diluted solutions and thus certainly containing a lower amount of nutrients, the reduced alkalinity might have increased the availability of these nutrients. In particular, the shoots of the 1:90 plants contained much more Cu (+97% and +58%, respectively) and Zn (+272 and 23%, respectively) compared with the shoots of the 1:30 and 1:60 plants (Figure 5). Consequently, since nutrients provide essential building blocks for plant growth, the higher accumulation of these nutrients in 1:60 and 1:90 maize plants could explain their higher biomass accumulation compared with the 1:30 plants. In addition, the growth of the 1:30 plants was further hindered by the additive toxic effects of Na-B interaction. Both elements were not translocated to the leaves but were stored defensively in the roots (Figure 3C; Ismail, 2003).

## Conclusion

The results presented here contribute to expanding the current rather scarce knowledge on the composition and level of phytotoxic substances in AHL as a consequence of the type of feedstock and thermochemical reactions of the HTC process. In addition, the physiological responses of maize plants, when grown at different AHL dilution ratios, have been also evidenced. On the one hand, the 1:30 AHL solution was too rich in potentially phytotoxic substances (mainly Na) and with a very alkaline pH that reduced the bioavailability of the nutrients and thus led to growth arrest. On the other, the 1:60 and 1:90 solutions were very similar in terms of composition and impact on plant growth. In particular, while the less concentrated phytotoxicity load, which allowed plant growth, has to be considered an advantage, the levels of nutrient concentration are surely a disadvantage, because they hindered the proper photosynthetic functionality, as demonstrated by the more pronounced chlorosis appearance and more reduced chlorophyll content.

Therefore, it is necessary to carry out further research to identify the right dilution ratio, which represents a good compromise between not causing phytotoxicity damage and providing enough amounts of essential nutrients for proper plant growth and development. In addition, *ad-hoc* treatments of Na limitation or removal are absolutely required before using AHL as a fertilizer solution. Finally, given the high genetic variation between plant species, future studies are undoubtedly needed to identify the most suitable species for growth in fertilizer solutions composed partly of AHLs by the HTC process. As an example, the aptitude of tomato to grow and produce even under high-salt conditions makes this species a good candidate.

## Data Availability Statement

The original contributions presented in the study are included in the article/**Supplementary Material**, further inquiries can be directed to the corresponding authors.

## Author Contributions

SCl and TM designed the study. SCl, ML, AB, and VB performed the experiments, and collected and analyzed the data. SCl wrote the original draft of the manuscript. VB contributed to the manuscript preparation. VB, MB, SCs, and TM revised and edited the original draft of the manuscript. DB, MB, SCs, and TM supervised and acquired the funds. All the authors gave their final approval of the submitted version.

### Conflict of Interest

DB was employed by the company HBI S.r.l., Bolzano, Italy.

The remaining authors declare that the research was conducted in the absence of any commercial or financial relationships that could be construed as a potential conflict of interest.

## Footnotes

^1^https://www.biogas-wipptal.com/en/
