# Supplementary material for: Evaluating the Aqueous Phase From Hydrothermal Carbonization of Cow Manure Digestate as Possible Fertilizer Solution for Plant Growth
Source: Front Plant Sci. 2021 Jun 30;12:687434. doi: 10.3389/fpls.2021.687434 (PMC8278309; doi:10.3389/fpls.2021.687434)
Supplement: Supplementary file 1 [file Table_1.DOCX]

| **Mineral elements_D_** | |  |
| --- | --- | --- |
| B (µg g_DW_^-1^) | 49.8 ± 2.6 | |
| Ca (mg g_DW_^-1^) | 13.0 ± 0.3 | |
| Cd ( – ) | < LOD | |
| Co (µg g_DW_^-1^) | 0.4 ± 0.1 | |
| Cr (µg g_DW_^-1^) | 4.7 ± 0.2 | |
| Cu (µg g_DW_^-1^) | 48.5 ± 0.9 | |
| Fe (mg g_DW_^-1^) | 1.4 ± 0.1 | |
| Mg (mg g_DW_^-1^) | 5.0 ± 0.1 | |
| Mn (µg g_DW_^-1^) | 148.1 ± 3.9 | |
| Mo (µg g_DW_^-1^) | 2.3 ± 0.1 | |
| Na (mg g_DW_^-1^) | 6.7 ± 0.1 | |
| Ni (µg g_DW_^-1^) | 3.6 ± 0.1 | |
| P (mg g_DW_^-1^) | 4.8 ± 0.1 | |
| Pb (µg g_DW_^-1^) | 1.5 ± 0.1 | |
| S (mg g_DW_^-1^) | 3.0 ± 0.1 | |
| Zn (µg g_DW_^-1^) | 140.2 ± 3.4 | |

**Supplementary Table 1.** Concentration values of main mineral elements of the digestate (D) reported in Fig. 1B: boron (B), calcium (Ca), cadmium (Cd), cobalt (Co), chrome (Cr), copper (Cu), iron (Fe), magnesium (Mg), manganese (Mn), molybdenum (Mo), sodium (Na), nickel (Ni), phosphorus (P), lead (Pb), sulfur (S), and zinc (Zn). DW = dry weight; LOD = limit of detection. The LOD of Cd by ICP-OES was 0.177 µg L^-1^. Data are presented as means ± SE (n = 3).

**Supplementary Table 2.** Concentration values of main mineral elements of the aqueous HTC liquid (AHL) reported in Fig. 2B: boron (B), calcium (Ca), cadmium (Cd), cobalt (Co), chrome (Cr), copper (Cu), iron (Fe), magnesium (Mg), manganese (Mn), molybdenum (Mo), sodium (Na), nickel (Ni), phosphorus (P), lead (Pb), sulfur (S), and zinc (Zn). LOD = limit of detection. The LODs of Cd and Pb by ICP-OES were 0.177 and 6.730 µg L^-1^, respectively. Data are presented as means ± SE (n = 3).

| **Mineral elements_AHL_** |  |
| --- | --- |
| B (mg L^-1^) | 16.2 ± 0.2 |
| Ca (mg L^-1^) | 188.9 ± 86.8 |
| Cd ( – ) | < LOD |
| Co (µg L^-1^) | 33.9 ± 4.5 |
| Cr (µg L^-1^) | 72.4 ± 38.9 |
| Cu (mg L^-1^) | 1.1 ± 0.3 |
| Fe (mg L^-1^) | 20.8 ± 8.3 |
| Mg (mg L^-1^) | 61.0 ± 30.8 |
| Mn (mg L^-1^) | 2.1 ± 0.9 |
| Mo (µg L^-1^) | 570.0 ± 5.0 |
| Na (mg L^-1^) | 628.3 ± 34.2 |
| Ni (µg L^-1^) | 546.0 ± 49.1 |
| P (mg L^-1^) | 101.2 ± 29.6 |
| Pb ( – ) | < LOD |
| S (mg L^-1^) | 200.3 ± 10.1 |
| Zn (mg L^-1^) | 2.2 ± 1.0 |
